# Relationship between Distance from Major Roads and Adolescent Health in Japan

**DOI:** 10.2188/jea.12.418

**Published:** 2007-11-30

**Authors:** Yoshihiro Miyake, Akiko Yura, Masayuki Iki

**Affiliations:** 1 Department of Public Health, Kinki University School of Medicine.

**Keywords:** road traffic, allergy, cross-sectional studies, children

## Abstract

A relationship between distance from major roads and the prevalence of allergic disorders and general symptoms among junior high school students was assessed, separating the effects of distance of residence and school from the roads. Study subjects were 5,652 students aged 12 to 15 years. This study used diagnostic criteria from the International Study of Asthma and Allergies in Childhood. The questionnaire also asked about symptoms of headache, stomachache, tiredness, and cough and the shortest distance from residence to major roads. Distance from school to the nearest major road was measured on a map. Adjustment was made for gender, grade, the number of older siblings, smoking in the household, and maternal history of allergy. A shorter distance between residence and major roads was associated with an increased prevalence of headache, stomachache, tiredness, and cough. There was a marginally significant positive association between residence facing major roads and the prevalence of allergic rhinoconjunctivitis. Residence within 100 m of major roads showed a tendency for a positive relationship with the prevalence of wheeze and atopic dermatitis. There was no apparent relationship between distance of school from major roads and allergic disorders or the general symptoms. The findings suggest that proximity of residence, not school, to major roads may be associated with an increased prevalence of allergic disorders, headache, stomachache, and tiredness among Japanese adolescents. Further investigations with more precise and detailed exposure and health outcome measurements are needed to corroborate the relationship between traffic related factors and allergic disorders and general symptoms.

Although a possible relationship between road traffic and respiratory symptoms and allergic disorders has been investigated in a number of epidemiologic studies,^1^^-^^10^ it remains uncertain whether road traffic is related to an increased prevalence of such symptoms. Several Japanese studies have shown a positive relationship between distance of residence from major roads and prevalence of respiratory symptoms.^1^^-^^4^ Another study in Japan reported an increased prevalence of cedar pollinosis among residents located contiguous to the inter-city roads where old cedar trees were prevalent.^5^ A cross-sectional study of Germanic schoolchildren showed a positive association between indicators of traffic density and the prevalence of wheezing as well as allergic rhinitis.^6^ Another study in Germany reported an association between the maximum traffic counts in the school districts and childhood respiratory symptoms. A small increase in odds ratio was observed for respiratory infections and the prevalence of wheezing in this study, while there were no differences in the prevalence of asthma and hay fever.^7^ A British case-control study showed a significant positive association between road traffic and risk of hospital admission for asthma in children younger than 5 years of age.^8^ However the association was not found in a case-control study of UK children aged 5-14 years.^9^ A study in East and West Germany found a significant positive association between residence closer than 50 m from a busy road and the prevalence of atopic eczema.^10^

In Japan, the prevalence of general symptoms such as headache and tiredness has been increasing among schoolchildren.^11^ It is hypothesized that changes in the living environment and disordered diet and life style may be responsible for the increase of these symptoms.^12^ To our knowledge, no studies have examined the effect of road traffic on such symptoms.

Schoolchildren spend much of their time at or near their home and school addresses. We assessed the relationship between the distance from major roads and the prevalence of allergic disorders and general symptoms in a cross-sectional study of 5,652 Japanese adolescents, separating the effects of distance of residence and school from the roads.

## MATERIALS AND METHODS

Suita City, with a total population of almost 350,000, is a suburban residential city adjacent to Osaka City, the largest city in western Japan, and is served by an extensive and busy network of highways and major roads with severe traffic congestion. All of the 18 public junior high schools in Suita City participated in surveys of general medical histories and subjective symptoms conducted by the Suita City Municipal Board of Education during the period of October 22 through 26, 2001. A questionnaire, based on a health symptom questionnaire used in the Children’s Health Survey in Osaka Prefecture,^11^ was distributed by the teachers to all of the 9,008 junior high school children aged 12-15 years, and 6,845 questionnaires (76%) answered by their parents were returned. A total of 1,193 children were excluded for missing or implausible data on the exposures, outcomes, and confounding factors under study. The final analysis included 5,652 subjects (63%).

The questionnaire included core questions used in the International Study of Asthma and Allergies in Childhood regarding symptoms of wheeze, atopic eczema, and rhinoconjunctivitis in the last 12 months, which have been reported in details elsewhere.^13^^-^^16^

The questionnaire also asked about gender, grade, the number of older siblings, shortest distance of residence from one of the six major roads, smoking in the household, and maternal history of asthma, atopic dermatitis, and allergic rhinitis. The six major roads included an expressway, the Meishin Expressway, two national highways, Route 423 and Route 479, and three major Osaka Prefectural roads, the Osaka Central Loop, the Osaka-Takatsuki-Kyoto Line, and the Ibaragi-Settsu Line, defined as having maximal daytime traffic volume of 20,000 vehicles or more per 12 hours.^17^ Distance of school buildings from the nearest major road was measured on a map. Headache, stomachache, and tiredness were defined as present if the child had often complained of the respective symptoms before entry into the study. Cough was defined as frequent coughing without cold. Smoking in the household was defined as positive if 10 or more cigarettes a day were smoked inside the house. Maternal history of asthma, atopic dermatitis, and allergic rhinitis (including cedar pollinosis) were considered present if the mother had experienced each of these disorders before entry into the study.

Distance of residence from major roads was classified into three categories (facing, <100 m, and 100+ m); distance of school from major roads into two (<100 m and 100+ m); and the number of older siblings into three (0, 1, and 2+). Logistic regression analysis was used to estimate crude odds ratios (ORs) and their 95% confidence intervals (CIs) for these factors. Multiple logistic regression analysis was used to control for the confounding effects of selected factors. Trend of association was assessed by a logistic regression model assigning scores to the levels of the independent variable. Two-sided p values less than 0.05 were regarded as statistically significant. All computations were done by the PC-SAS version 6.12 (SAS Institute, Inc., Cary, NC, USA).

## RESULTS

As shown in Table 1, the overall prevalence values for symptoms of wheeze, atopic eczema, and rhinoconjunctivitis in the last 12 months were 6.9%, 14.6%, and 24.4%, respectively. The corresponding prevalence of headache, stomachache, tiredness, and cough were 13.2%, 24.3%, 29.6%, and 2.9%, respectively.

**Table 1.** Prevalence of allergic disorders in the nearest 12 months and general symptoms in 5,652 children, Suita, Japan, 2001.

Allergic disorders	No. (%)	
Wheeze	388	(6.9)
Atopic dermatitis	828	(14.6)
Allergic rhinoconjunctivitis	1,377	(24.4)
Headache	748	(13.2)
Stomachache	1,376	(24.3)
Tiredness	1,673	(29.6)
Cough	164	(2.9)

The distribution of selected factors under study in 5,652 children is summarized in Table 2. About a half of the children had no siblings and at least one smoker in the household. There were many more children with a maternal history of allergic rhinitis than those with a maternal history of asthma or atopic eczema. Only 7% of residences fronted onto major roads, while 78% of children lived more than 100 m from major roads and 40% attended six of 18 public junior high schools within 100 m of major roads.

**Table 2.** Distribution of selected factors in 5,652 children, Suita, Japan, 2001.

Factors	No. (%)	
Male sex	2,749	(48.6)
Grade in junior high schools		
1	1,959	(34.7)
2	1,901	(33.6)
3	1,792	(31.7)
Number of older siblings		
0	2,656	(47.0)
1	2,180	(38.6)
2 +	816	(14.4)
Smoking in household	2,700	(47.8)
Maternal history of asthma	363	(6.4)
Maternal history of atopic dermatitis	328	(5.8)
Maternal history of allergic rhinitis	2,474	(43.8)
Distance of residence from major roads		
Facing	372	(6.6)
< 100 m	887	(15.7)
100 + m	4,393	(77.7)
School within 100 m of major roads	2,234	(39.5)

Table 3 presents the relationship between distance from major roads and the prevalence of allergic disorders in terms of crude and adjusted ORs and 95% CIs, separating the effects of distance of residence and school from the roads. Little difference was found between crude and adjusted ORs for each disorder. After controlling for sex, grade, older siblings, smoking in the household, and maternal history of asthma, atopic dermatitis, and allergic rhinitis, residence within 100 m of major roads showed a tendency for a positive relationship with the prevalence of wheeze and atopic dermatitis while there was no substantial increase in the prevalence for residence facing major roads. Residence facing major roads was marginally associated with an increased prevalence of allergic rhinoconjunctivitis (adjusted OR = 1.27, 95% CI: 1.00-1.62). An association between residence within 100 m of major roads and allergic rhinoconjunctivitis was not evident, however. No relationship between distance of school from major roads and these allergic disorders was found.

**Table 3.** Odds ratios (ORs) and their 95% confidence intervals (CIs) of allergic disorders according to distance of residence and school from major roads in 5,652 children, Suita, Japan, 2001.

Distance from major roads	Residence		School	
	
	Crude OR (95% CI)	Adjusted OR (95% CI)*	Crude OR (95% CI)	Adjusted OR (95% CI)*
Wheeze				
Facing	1.08 (0.69-1.60)	1.08 (0.69-1.61)		
< 100 m	1.32 (1.01-1.71)	1.27 (0.97-1.66)	1.17 (0.95-1.44)	1.11 (0.90-1.37)
100 + m	1.0	1.0	1.0	1.0
p for trend^+^	0.17	0.23		
Atopic dermatitis				
Facing	0.93 (0.68-1.26)	0.92 (0.67-1.25)		
< 100 m	1.24 (1.02-1.51)	1.22 (1.00-1.48)	1.09 (0.94-1.27)	1.07 (0.92-1.24)
100 + m	1.0	1.0	1.0	1.0
p for trend^+^	0.40	0.52		
Allergic rhinoconjunctivitis				
Facing	1.27 (1.00-1.60)	1.27 (1.00-1.62)		
< 100 m	1.04 (0.88-1.23)	1.00 (0.84-1.19)	1.07 (0.95-1.21)	1.05 (0.92-1.19)
100 + m	1.0	1.0	1.0	1.0
p for trend^+^	0.07	0.12		

* Based on multiple logistic regression controlling for sex, grade (1, 2, and 3), older siblings (0, 1, and 2 +), smoking in household (< 10 and 10 + cigarettes a day smoked inside the house), distance of residence from major roads, distance of school from major roads, and maternal history of asthma, atopic dermatitis, and allergic rhinitis.

^+^ Based on logistic regression with scores 0-2 assigned to the three levels.

The results for general symptoms are showed in Table 4. After adjustment for the potential confounding factors, residence within 100 m of major roads was independently associated with an increased prevalence of headache (adjusted OR = 1.41, 95% CI: 1.15-1.72) and tiredness (adjusted OR = 1.27, 95% CI: 1.08-1.48), showing a positive dose-response relationship (p for linear trend = 0.0009 and 0.02, respectively). Such a positive relationship was also observed for stomachache (p for linear trend = 0.04), but the ORs comparing residence facing major roads and residence within 100 m with residence more than 100 m from major roads were not statistically significant (adjusted OR = 1.26, 95% CI: 0.99-1.59 and 1.10, 95% CI: 0.93-1.31, respectively). Shorter distance of residence from major roads was associated with a 1.3-fold increased prevalence of cough, but the estimation was not statistically significant as indicated by a wide 95% CI. There were no material relations between distance of school from major roads and the prevalence of the four general symptoms.

**Table 4.** Odds ratios (ORs) and their 95% confidence intervals (CIs) of general symptoms according to distance of residence and school from major roads in 5,652 children, Suita, Japan, 2001.

Distance from major roads	Residence		School	
	
	Crude OR (95% CI)	Adjusted OR (95% CI)*	Crude OR (95% CI)	Adjusted OR (95% CI)*
Headache				
Facing	1.40 (1.04-1.85)	1.36 (1.01-1.81)		
< 100 m	1.40 (1.15-1.71)	1.41 (1.15-1.72)	0.93 (0.80-1.09)	0.91 (0.77-1.07)
100 + m	1.0	1.0	1.0	1.0
p for trend^+^	0.0004	0.0009		
Stomachache				
Facing	1.28 (1.01-1.61)	1.26 (0.99-1.59)		
< 100 m	1.10 (0.93-1.29)	1.10 (0.93-1.31)	0.92 (0.81-1.04)	0.91 (0.80-1.03)
100 + m	1.0	1.0	1.0	1.0
p for trend^+^	0.03	0.04		
Tiredness				
Facing	1.15 (0.91-1.44)	1.13 (0.89-1.41)		
< 100 m	1.28 (1.09-1.49)	1.27 (1.08-1.48)	1.02 (0.91-1.15)	1.01 (0.89-1.13)
100 + m	1.0	1.0	1.0	1.0
p for trend^+^	0.01	0.02		
Cough				
Facing	1.30 (0.69-2.24)	1.33 (0.71-2.30)		
< 100 m	1.34 (0.89-1.97)	1.32 (0.87-1.95)	1.14 (0.83-1.56)	1.10 (0.80-1.51)
100 + m	1.0	1.0	1.0	1.0
p for trend^+^	0.15	0.14		

* Based on multiple logistic regression controlling for sex, grade (1, 2, and 3), older siblings (0, 1, and 2 +), smoking in household (< 10 and 10 + cigarettes a day smoked inside the house), distance of residence from major roads, distance of school from major roads, and maternal history of asthma, atopic dermatitis, and allergic rhinitis.

^+^ Based on logistic regression with scores 0-2 assigned to the three levels.

## DISCUSSION

The present study showed that shorter distance of residence from major roads was associated with an increased prevalence of headache, stomachache, tiredness, and cough. There was a marginally significant positive association between residence facing major roads and the prevalence of allergic rhinoconjunctivitis. Residence within 100 m of major roads was positively related to the prevalence of wheeze and atopic dermatitis, although the relation was not statistically significant. There was no apparent relationship between distance of school from major roads and allergic disorders or the general symptoms under study.

Shorter distance from major roads may substitute as an indicator for automobile exhaust as a risk factor for allergic disorders and general symptoms. A study in Tokyo reported that there was obvious gradient for nitrous oxide (NO) according to the distance from the roadside and that the concentrations of nitrogen oxide (NO_2_) and suspended particle matter at 0 m were slightly higher than those at 150 m from the roadside.^3^ The previous cited study showed that NO and NO_2_ concentrations along Kannana-dori Avenue varied among sites.^3^ However, in Suita City, information was not available regarding the relationship between the distance from major roads and ambient pollution concentrations. Traffic volumes were distributed over a wide range among six major roads in this study. Thus, the present study had substantial limitations with respect to categorization of the distance from major roads.

A panel study in Mexico City, where ambient measures of particular matter and ozone frequently exceeded the Mexican standards for these contaminants, demonstrated that an increase of these air pollutants was significantly associated with an increase in lower respiratory symptoms among children with mild asthma.^18^ Another panel study of children in the Netherlands found that only the group with bronchial hyperresponsiveness and relatively high concentrations of serum total IgE appeared susceptible to air pollution.^19^ These observations suggest interactive effects between air pollutants and allergic responses. A Japanese study observed an increased prevalence of cedar pollinosis in areas with heavy traffic combined with high levels of cedar pollen.^5^ Exposure to cedar pollen may be ubiquitous in the whole country irrespective of distance from cedar trees, because the government encouraged the planting of cedar trees for the construction of housing after World War II. In this study, the joint effect of cedar pollen and air pollutants may have contributed to the increased clinical manifestation of allergic rhinoconjunctivitis among children residing along major roads. Very young children may be more susceptible to the effects of air pollution than older children. In two British case-control studies,^8^^,^^9^ a significant positive association between road traffic and asthma was found in children younger 5 years, but not those aged 5-14 years.

We have no clear explanation as to underlying mechanisms for the positive relationship between shorter distance of residence from major roads and the prevalence of headache, stomachache, tiredness, and cough. The positive relationships may be more attributable to noises, vibrations, excessive light during the nighttime, or psychological stress in relation to major roads rather than air pollutants. It is not likely that children living within 100 m of major roads differ from those living more than 100 m in several aspects such as disordered lifestyle and physical inactivity. Nonetheless, part of the positive relationships may be ascribed to such uncontrolled confounders.

In the present study, distance of school from major roads was unrelated to the prevalence of all symptoms under study. In Japan, students go to junior high school for three years. A longer period of exposure to major roads may be necessary to show a significant positive association between road traffic and these symptoms.

The present study had several methodological advantages. The prevalence of allergic disorders was assessed by validated core questions in the International Study of Asthma and Allergies in Childhood Phase One study. The number of study subjects was fairly large and subjects were homogenous in terms of age and residential background. Weaknesses of this study should be borne in mind, however. Of a total of 9,008 public junior high school students in Suita City, 5,652 (63%) were included in the study. It is difficult to know whether the exclusion of the subjects was selective concerning exposure, outcome, or both. However, there was no material difference between participants and non-participants with respect to sex and grade. Study subjects were adolescent children who lived an urban life. Moreover, the prevalence values of allergic disorders observed in the present population was not equal to those reported for Japanese children in the International Study of Asthma and Allergies in Childhood.^13^^-^^15^ Thus, it may be difficult to generalize the present findings. Within multifactorial pathogenesis, several contributing environmental factors have been linked to allergic disorders. However, no allowance was made for external factors such as aeroallergens, food allergens, and especially indoor pollution. Information on distance of residence from major roads was taken from self-reports of the child’s parents. However, it seems probable that all subjects had the same probability of being misclassified in relation to their exposure status. The consequence, if such hypothetical misclassification occurred, would have given rise to an underestimation of our findings. The definition of symptoms of headache, stomachache, tiredness, and cough was crude and may have overestimated children with these symptoms because of the unreliable nature of children’s reporting. If the child’s parents were not aware of the possible ill effects of proximity to major roads, misclassification of the outcomes was not likely to be different among all categories of distance from major roads. It also introduced a bias toward the null.

This is the first epidemiologic study on the relationship between distance from major roads and the prevalence of allergic disorders, headache, stomachache, and tiredness in Japanese adolescents. Further investigations with more precise and detailed exposure and health outcome measurements are needed to draw a conclusion as to the question of whether traffic related factors are independent risk factors for these symptoms.
